# Three Dimensional Identification of Medial and Lateral Vestibulospinal Tract in the Human Brain: A Diffusion Tensor Imaging Study

**DOI:** 10.3389/fnhum.2018.00229

**Published:** 2018-06-05

**Authors:** Sung H. Jang, Jung W. Kwon, Sang S. Yeo

**Affiliations:** ^1^Department of Physical Medicine and Rehabilitation, College of Medicine, Yeungnam University, Daegu, South Korea; ^2^Department of Physical Therapy, College of Health Sciences, Dankook University, Cheonan, South Korea

**Keywords:** vestibulospinal tract, vestibular nuclei, balance, gait, diffusion tensor imaging

## Abstract

**Purpose:** The vestibulospinal tract (VST) is involved in balance control and gait function. No research has identified the VST in the human brain. In the current study, we attempted to identify the medial and lateral VST in the human brain, using diffusion tensor tractography (DTT).

**Materials and Methods:** We recruited 40 healthy volunteers for this study. For reconstruction of the medial VST, a seed region of interest (ROI) was placed on the medial vestibular nuclei in the pons and target ROI on the posteromedial medulla. For reconstruction of the lateral VST, a seed ROI was placed on the lateral vestibular nuclei of pons and the target ROI on the posterolateral medulla. Values of fractional anisotropy (FA), mean diffusivity (MD), and tract volume of the medial and lateral VST were measured.

**Results:** The medial VST, which originates from the medial vestibular nuclei, descends through the posteromedial medulla, and terminates at the anterior funiculus of the cervical spinal cord. The lateral VST originates from the lateral vestibular nuclei, and terminates in the anterior portion of lateral funiculus, through the posterolateral medulla. The FA value of medial VST was significantly higher than that of lateral VST. In contrast, the MD value and tract volume were significantly lower than those of lateral VST (*p* < 0.05).

**Conclusion:** We identified the medial and lateral VST in the human brain using DTT and investigated the anatomical characteristics of the medial and lateral VST. The methodology and results of this study could be helpful to both clinicians and researchers in the neuroscience field.

## Introduction

The vestibulospinal tract (VST) is an extrapyramidal motor pathway for control of balance in gait (Markham, 1987; Green and Angelaki, 2010; Highstein and Holstein, 2012; Zorner et al., 2014; Jung et al., 2015; Lambert et al., 2016). The vestibulocochlear nerves send information to the vestibular nuclei about changes in the orientation of the head, and vestibular nuclei transmit motor commands to maintain balance of upright posture of body and head through the VST (Sadjadpour and Brodal, 1968; Markham, 1987; Afifi and Bergman, 2005; Green and Angelaki, 2010; Highstein and Holstein, 2012; Zorner et al., 2014; Jung et al., 2015; Lambert et al., 2016). The VST is classified into two sub-pathways; the medial VST originates from the medial vestibular nuclei and connects to the anterior funiculus of the upper cervical spinal cord, and the lateral VST originates in the lateral vestibular nuclei and terminates at the lateral funiculus through total length of spinal cord (Sadjadpour and Brodal, 1968; Nathan et al., 1996; Afifi and Bergman, 2005; Gullapalli et al., 2006; Lin and Bono, 2010). Given the importance of the VSTs in balance and gait function (Markham, 1987; Ferber-Viart et al., 1999; Green and Angelaki, 2010; Highstein and Holstein, 2012; Curthoys et al., 2014; Papathanasiou et al., 2014; Zorner et al., 2014; Jung et al., 2015; Lambert et al., 2016), anatomical identification of the VST could provide useful information for the neuroscience field. However, studies in this area of live human brain research are very limited.

Recent developments in diffusion tensor tractography (DTT), derived from diffusion tensor imaging (DTI), allow visualization and localization of neural tracts at the subcortical level in three dimensions (Mori et al., 1999; Assaf and Pasternak, 2008). Many DTT studies identify and visualize the pyramidal and extrapyramidal tracts in the human brain such as the corticospinal tract, rubrospinal tract and corticoreticulospinal tract, and so on (Han et al., 2010; Hong et al., 2010; Yang et al., 2011; Yeo et al., 2012; Jang et al., 2013). However, no study of the medial and lateral VST has been reported.

In the current study, we attempted to identify and investigate the anatomical characteristics of the medial and lateral VST in human brain, using DTT. The clinical application for the current study is its possible use for prognostic and therapeutic purposes among patients with brain injury who showed significant balance problem or central vestibular disorder.

## Materials and Methods

### Subjects

Forty normal healthy subjects (23 males, 17 females; mean age, 36.2 ± 9.5 years; range, 20–50) with no history of neurologic disease were recruited for the study. All participants provided written informed consent; the study was approved by the institutional review board at our hospital.

### Diffusion Tensor Image

Acquisition of DTI data was performed using a 6-channel head coil on a 1.5 T Philips Gyro scan Intera (Philips, Best, Netherlands) and single-shot echo-planar imaging. For each of the 32 non-collinear diffusion sensitizing gradients, 67 contiguous slices were acquired parallel to the anterior commissure-posterior commissure line. Imaging parameters were as follows: acquisition matrix = 96 × 96; reconstructed matrix = 192 × 192; field of view = 240 m × 240 m; TR = 10,726 ms; TE = 76 ms; parallel imaging reduction factor (SENSE factor) = 2; EPI factor = 49; *b* = 1000 s/mm^2^; NEX = 1; and a slice thickness of 2.5 mm with no gap (acquired voxel size 1.3 m × 1.3 m × 2.5 m).

### Probabilistic Fiber Tracking

Diffusion-weighted imaging data were analyzed using the Oxford Centre for Functional Magnetic Resonance Imaging of the Brain (FMRIB) Software Library (FSL^1^). Affine multi-scale two-dimensional registration was used to correct head motion effect and image distortion due to eddy current. Fiber tracking used a probabilistic tractography method based on a multifiber model, and applied in the present study utilizing tractography routines implemented in FMRIB Diffusion (5000 streamline samples, 0.5 mm step lengths, curvature thresholds = 0.2).

**FIGURE 1:**
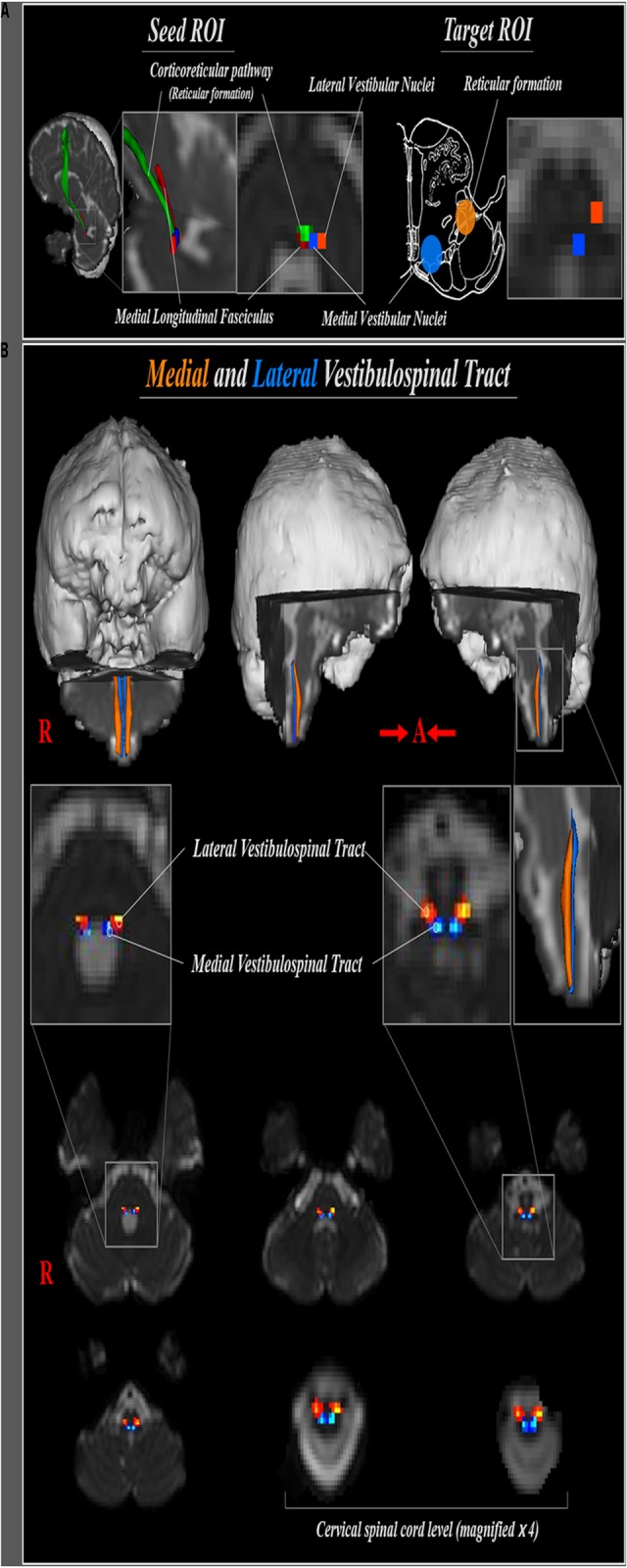
**(A)** Seed regions of interest (ROI) for medial and lateral vestibulospinal tract were placed on the medial (blue) and lateral (orange) vestibular nuclei at the level of pons, respectively. Target ROI for medial and lateral vestibulospinal tract were placed on the medial vestibular nuclei (blue) and reticular formation (orange), respectively. **(B)** The neural pathways of medial (blue) and lateral (orange) vestibulospinal tract between pontine vestibular nuclei and upper cervical spinal cord are shown at each brain level in a normal subject (a 33-year-old male).

The medial VST was determined by selection of fibers passing through seed and two target regions of interest (ROI) (**Figure 1A**). The medial VST originates in the medial vestibular nuclei, Schwalbe’s nuclei, in the pons and medulla level, and terminates in the anterior funiculus of the cervical spinal cord (Sadjadpour and Brodal, 1968; Nathan et al., 1996; Afifi and Bergman, 2005; Lin and Bono, 2010; Kirsch et al., 2016). Therefore, we determined the seed ROI as the medial vestibular nuclei in the caudal portion of pons (anterior boundary: pontine reticular formation; posterior boundary: forth ventricle; medial boundary: medial longitudinal fasciculus; lateral boundary: lateral vestibular nuclei) and the target ROI on the posteromedial medulla, corresponding to medial vestibular nuclei in medulla. The lateral VST originates in the Deiters’ nucleus or lateral vestibular nuclei of the pons, and descends through the reticular formation of medulla to lateral funiculus of spinal cord (Sadjadpour and Brodal, 1968; Nathan et al., 1996; Afifi and Bergman, 2005; Lin and Bono, 2010; Sakaie et al., 2011; Yeo et al., 2012; Kirsch et al., 2016). Therefore, for analysis of the lateral VST, the seed ROI was placed on the lateral vestibular nuclei at the level of pons (anterior boundary; inferior cerebellar peduncle; posterior boundary: dentate nucleus; medial boundary: medial vestibular nuclei; lateral boundary: middle cerebellar peduncle), and the target ROI on the posterolateral medulla corresponding to the reticular formation of the medulla. Out of 5000 samples generated from the seed voxel, results were visualized at the threshold of 1 streamline through each voxel for analysis. Values of fractional anisotropy (FA), mean diffusivity (MD), and tract volume of the medial and lateral VST were measured.

### Statistical Analysis

SPSS software (v.20.0; SPSS, Inc., an IBM Company, Chicago, IL, United States) was used for data analysis. The independent *t*-test was used for determination of the difference of values of DTI parameters between medial and lateral VST, and between right and left hemispheres. Null hypotheses of no difference were rejected if *p*-values were less than 0.05.

## Results

The reconstructed medial VST, which originates from the medial vestibular nuclei at the level of lower pons, descends through postero-medial region of medulla oblongata, then terminates at the anterior funiculus of the spinal cord. The lateral VST originates from the lateral vestibular nuclei in the lower pons, and descends through the antero-lateral region of the medulla oblongata and lateral funiculus of spinal cord (**Figure 1B**).

The mean value for FA was 0.44 ± 0.06, for MD 0.98 ± 0.12, and for tract volume of medial VST 241.37 ± 75.06. In the lateral VST, the mean FA value was 0.40 ± 0.05, for MD 1.18 ± 0.19, and for tract volume was 361.20 ± 92.99. In terms of FA value, medial VST was significantly higher than lateral VST (*p* < 0.05). In contrast, MD value and tract volume were significantly lower than lateral VST (*p* < 0.05). No DTI parameters of the medial and lateral VST differed between right and left hemisphere (*p* > 0.05) (**Table 1**).

**Table 1:** Comparison of diffusion tensor parameters between the medial and lateral vestibulospinal tract.

	Medial vestibulospinal tract			Lateral vestibulospinal tract			*p*
	Right	Left	Total	Right	Left	Total	
FA	0.44 (0.06)	0.43 (0.06)	0.44 (0.06)	0.40 (0.05)	0.44 (0.06)	0.40 (0.05)	0.004^∗^
MD	0.98 (0.11)	0.99 (0.13)	0.98 (0.12)	1.15 (0.15)	0.98 (0.12)	1.18 (0.19)	0.000^∗^
Tract volume	227.80 (60.05)	254.93 (87.58)	241.37 (75.06)	385.60 (108.37)	336.80 (69.95)	361.20 (92.99)	0.000^∗^

*Values represent mean ( ± standard deviation). FA, fractional anisotropy; MD, mean diffusivity, MD × 10^-3^(mm^2^/s). Independent-test was used to compare the difference of total diffusion tensor imaging parameters between medial and lateral vestibulospinal tract. ^∗^p < 0.05*.

## Discussion

In the current study, we reconstructed the medial and lateral VST from the pontine vestibular nuclei to the upper cervical spinal cord in normal subjects using DTT. The reconstructed medial and lateral VST originate from the medial and lateral vestibular nuclei, respectively. In the medulla, the medial VST passes through the posteromedial medulla, corresponding to medial vestibular nuclei, and terminates at the anterior funiculus of the cervical spinal cord. Conversely, the lateral VST passes through the posterolateral medulla, corresponding to the reticular formation, then terminates at the anterior portion of lateral funiculus cervical spinal cord. As for the course of the medial and lateral VST, our results appear to agree with animal studies (Sadjadpour and Brodal, 1968; Suzuki, 1985; Gullapalli et al., 2006; Dutheil et al., 2011; Zorner et al., 2014; Kasumacic et al., 2015). Regarding DTT parameters, the FA value of the medial VST was higher than the lateral VST, while the MD value and tract volume were the opposite. The FA value represents the degree of directionality of microstructures, and the MD value indicates the magnitude of water diffusion (Mori et al., 1999; Assaf and Pasternak, 2008). In contrast, the tract volume was determined by the number of voxels contained within a neural tract (Mori et al., 1999; Assaf and Pasternak, 2008). Therefore, high FA value with low MD value may indicate high directionality and low tract volume indicate compact structure of medial VST compared to the lateral VST. In addition, we assumed that difference of DTT parameters between medial VST and lateral VST, high directionality and compact structure of medial VST, could be concerned with anatomical characteristic of each neural tract. Many previous studies suggested that the lateral VST is descending spinal tract through total length of spinal cord, in contrast, the medial VST is found only in the upper cervical spinal cord (Sadjadpour and Brodal, 1968; Nathan et al., 1996; Afifi and Bergman, 2005; Gullapalli et al., 2006; Lin and Bono, 2010).

Many studies have reported on the anatomical identification of the medial and lateral vestibular nuclei and VST in rat, cat and monkey brain (Sadjadpour and Brodal, 1968; Suzuki, 1985; Gullapalli et al., 2006; Dutheil et al., 2011; Zorner et al., 2014; Kasumacic et al., 2015). Furthermore, the locations of vestibular nuclei are same with the human and mammal brain, and the course of the medial and lateral VST in the human brain are essentially the same as the mammal brain (Collier and Buzzard, 1901). Nonetheless, accurate assessment of the medial and lateral VSTs were practically difficult, because the VST is not easy to discriminate from adjacent structures on conventional brain MRI. On the other hand, the neurophysiological assessment technique, the vestibular evoked myogenic potentials (VEMP), can estimate the function of VST in the human brain (Ferber-Viart et al., 1999; Curthoys et al., 2014; Papathanasiou et al., 2014; Jung et al., 2015; Piker et al., 2015). Although the VEMP efferent pathways travel through the pathways of VSTs (Ferber-Viart et al., 1999; Curthoys et al., 2014; Papathanasiou et al., 2014; Jung et al., 2015; Piker et al., 2015), the routes of the medial and lateral VST are not precisely classified (Ferber-Viart et al., 1999). Since introduction of DTI, one study has reported on the VST as far as we are aware. In 2006, using DTI, Gullapalli et al reported on the characteristics of longitudinal and transverse diffusivity of the VST in rat spinal cord (Gullapalli et al., 2006). They suggested that the VST in rat spinal cord showed highest longitudinal and transverse diffusivity compared with the corticospinal tract, reticulospinal tract and rubrospinal tract. Consequently, to our best knowledge, this is the first DTI study to identify the medial and lateral VST from the pontine vestibular nuclei to the spinal cord in human brain. However, several limitations of DTI should be considered. First, DTI may underestimate fiber tracts, and regions of fiber complexity and crossing can prevent full reflection of the underlying fiber architecture by DTI (Yamada et al., 2009). Second, we could not precisely define the location of ROIs because of the small and cramped size of vestibular nuclei. Third, we could not reconstruct the full length of the lateral VST.

## Conclusion

In conclusion, we reconstructed the medial and lateral VST in the human brain using DTT and investigated the anatomical characteristics of the medial and lateral VST. The methodology and results of this study would be helpful to both clinicians and researchers in the neuroscience field. In particular, the described courses of the medial and lateral VST should be useful to clinicians treating balance and gait function. Further studies on clinical correlation, and the reliability and validity of the medial and lateral VST will be needed in the near future.

## Disclosures

Financial disclosure statements have been obtained, and no conflicts of interest have been reported by the authors or by any individuals in control of the content of this article.

## Ethics Statement

We declare that all human and animal studies have been approved by the Medical Ethics Committee of the Yeungnam University Medical Center and have therefore been performed in accordance with the ethical standards laid down in the 1964 Declaration of Helsinki and its later amendments. We declare that all patients gave informed consent prior to inclusion in this study.

## Author Contributions

SJ conceiving and designing the study, funding, data acquisition, manuscript development, and manuscript writing. JK manuscript development, data acquisition, and manuscript writing. SY manuscript development, data acquisition, manuscript writing, and manuscript authorization.

## Conflict of Interest Statement

The authors declare that the research was conducted in the absence of any commercial or financial relationships that could be construed as a potential conflict of interest. The reviewer OT and handling Editor declared their shared affiliation.

## Footnotes

1 www.fmrib.ox.ac.uk/fsl
